# Visual-spatial ability is more important than motivation for novices in surgical simulator training: a preliminary study

**DOI:** 10.5116/ijme.56b1.1691

**Published:** 2016-02-21

**Authors:** Marcus Schlickum, Leif Hedman, Li Felländer-Tsai

**Affiliations:** 1 Department of Clinical Science, Intervention and Technology (CLINTEC), Division of Orthopedics and Biotechnology Karolinska Institutet, Stockholm, Sweden; 2 Department of Psychology, Umeå University, Umeå, Sweden

**Keywords:** Motivation, video games, surgical simulator, surgical training, visual-spatial ability

## Abstract

**Objectives:**

To investigate whether surgical simulation performance and previous video gaming experience would correlate with higher motivation to further train a specific simulator task and whether visual-spatial ability would rank higher in importance to surgical performance than the above. It was also examined whether or not motivation would correlate with a preference to choose a surgical specialty in the future and if simulator training would increase the interest in choosing that same work field.

**Methods:**

Motivation and general interest in surgery was measured pre- and post-training in 30 medical students at Karolinska Institutet who were tested in a laparoscopic surgical simulator in parallel with measurement of visual-spatial ability and self-estimated video gaming experience.  Correlations between simulator performance metrics, visual-spatial ability and motivation were statistically analyzed using regression analysis.

**Results:**

A good result in the first simulator trial correlated with higher self-determination index (r =-0.46, p=0.05) in male students. Visual-spatial ability was the most important underlying factor followed by intrinsic motivation score and finally video gaming experience (p=0.02, p=0.05, p=0.11) regarding simulator performance in male students. Simulator training increased interest in surgery when studying all subjects (p=0.01), male subjects (p=0.02) as well as subjects with low video gaming experience (p=0.02).

**Conclusions:**

This preliminary study highlights individual differences regarding the effect of simulator training on motivation that can be taken into account when designing simulator training curricula, although the sample size is quite small and findings should be interpreted carefully.

## Introduction

Minimal invasive surgery takes advantage of existing orifices of the body or small incisions as entry ports for surgical procedures in order to reduce surgical trauma. It is considered to be more difficult to learn than conventional surgery,^1^ which has spurred the development of surgical simulators. The most recent simulators use virtual-reality to create standardized and customizable scenarios. It has been shown repeatedly that virtual-reality surgical simulator training is an effective way of acquiring basic technical skills and that there is a transfer effect to real surgical tasks.^2^ Some of the critical aspects of simulation training described include provision of feedback, deliberate practice, training to proficiency, the opportunity to practice at varying levels of difficulty and the inclusion of both cognitive teaching and hands-on training.^3^ Despite evidence of simulators being an effective method of training, implementation has been slow. The use of narrow research scopes,^4^ lack of data to set proficiency levels^5^ and validated tests^6^ have been identified as obstacles to successful implementation. Since human barriers have been suggested as a limiting factor to increased simulator training, there is a need to alter cultures and motivation in hospital personnel and the medical profession^7^ as well as modern curricula for training professional surgical teams.^8^ Further, it has been shown that voluntary simulator training leads to minimal participation in a training curriculum, i.e., when given unrestricted access

to a training facility subjects simply don’t show up.^9^^, ^^10^In order to facilitate simulator training and to increase our knowledge of what works and why, several background factors important for surgical simulator performance have been identified such as visual-spatial ability,^11^ visual working memory^12^ and video gaming experience and practice.^13^ In a recent study investigating the correlation between theoretical knowledge and demonstrated simulator performance, we found that female medical students completing an endourological simulator task more efficiently passed the theoretical examination in the basic surgical sciences with significantly higher scores than females with low efficiency in the urological simulator.^14^ We concluded that there were most likely several explanations for this finding such as a lower amount of current video gaming experience as well as possible differences in motivation.^14^ Considering these findings in the light of the studies regarding low attendance among surgical trainees when given free simulator training facility access ^9^^, ^^10^ it would be interesting to examine the role of motivation for simulator training performance and general interest in surgery, and if certain factors may alter motivation. To our knowledge, this has previously not been investigated. Such research would hopefully contribute to optimization and implementation of surgical simulator training.

Self-determination theory^15^ (SDT) has become a major theory of human motivation in contemporary psychology.^16^ SDT suggests that behavioral regulations, in this case reasons for participating in simulator training, can be ordered on a continuum according to the extent to which motivation is self-determined (autonomous). Intrinsic motivation is one of several types of motivation that refers to performing an activity for itself and the pleasure and satisfaction following this activity. In SDT the concept of competence is composed, among other factors, by the feeling of being skilful when performing a task. By stating four hypotheses we sought to approach surgical simulator training from a SDT perspective in highly motivated surgical novices.  According to SDT, perceived proficiency is important for intrinsic motivation.^15^ Thus our first hypothesis was that:

   •   Better performance for surgical novices when completing a surgical training task would correlate with higher intrinsic motivation to train that specific task.

Furthermore, since several previous studies have highlighted the importance of visual-spatial ability for surgical simulation training, the second hypothesis was that:

   •   Visual-spatial ability would rank higher in importance to surgical performance than motivation and video gaming experience.

Since a high intrinsic motivation is characterized by the urge to do something out of one`s own will, the third hypothesis was that:

   •   Higher self-determination and intrinsic motivation would correlate with a preference to choose a surgical specialty in the future and simulator training would increase the interest in choosing that same work field.

Finally, because of the visual similarities between surgical simulation and certain video games, the final hypothesis was that:

   •   A higher video gaming experience would correlate to a higher intrinsic motivation to train a surgical simulator task.

## Methods

### Study design

To test the first hypothesis, motivation was measured in subjects who had been tested in a surgical simulator. Correlation between simulator performance and motivation was statistically analyzed. The second hypothesis was tested using multivariate ranking analysis of visual-spatial ability scores, pre-training motivation score, self-estimated video gaming experience and simulator performance. By measuring interest in surgery pre and post-training in a surgical simulator and correlating these scores to initial scores of motivation, the third hypothesis was tested. Finally, pre-training motivation was correlated to self-estimated video gaming experience to test the fourth hypothesis.

### Participants and sample size

Thirty surgical novices participated in the study: 12 females and 18 males. None of the students reported any previous experience from virtual-reality image guided surgical simulators. The study population was recruited among medical students at Karolinska Institutet. All students were enrolled on a voluntary basis following an open announcement opening up for the possibility to recruit a study population consisting of students with special interest in surgery.  Data was gathered between October 2012 and December 2013. The regional ethical committee approved the study. We enrolled highly motivated students since future surgeons would most likely be highly motivated and therefore the results more relevant among these subjects.

### Testing of background factors

Before arriving at the test location, all subjects filled in a questionnaire regarding, age, sex, previous simulator training and video gaming experience. Video gaming experience was graded on a Likert scale between 1-7 where 1 corresponded to never playing and 7 corresponded to playing every day. Subjects estimated their current and previous video gaming (age 13-18) on the scale. The questionnaire has been used in our previous studies.^11^^, ^^13^ Subjects also rated their interest in surgery as a future specialty and interest in future simulator training on a scale 1-5 where 1 corresponded to no interest at all and 5 corresponded to a very high interest.

In order to test visual-spatial ability, the mental rotations test, version A (MRT-A) was used and administrated as in previous studies.^11^^, ^^13^

### Simulator testing

All students were tested in the validated Minimal Invasive Surgery Trainer Virtual Reality, (MIST-VR) simulator.^17^ The task Manipulative diathermy medium has been used in many of our previous studies regarding cognitive background variables.^11^^,^^13^^,^^14^ In this specific task, subjects are supposed to grab a virtual ball with forceps using their right hand, touch it with forceps using their left hand, exert the left forceps and insert a diathermy instrument with what they are supposed to use on a virtual square that appears three times on the ball. During that procedure the ball must be fixated inside a virtual box. The procedure is then repeated starting with the left hand, for a total of three repetitions with each hand. After the procedure is completed the subjects are given a total score that is calculated based on completion time and number of errors such as colliding with the instruments, using diathermy in the wrong location etc.  A lower total score represents a better score, scores ranging within 0-700. All students were given a standardized oral instruction regarding the goals of the task to be performed, how it should be performed in a correct way as well as what defined the score. The same instructor was in charge during all test occasions (MS). After the standardized instruction all students had one first try, followed by a training period of 30 minutes that ended in one last try. Before the last try the subjects were informed that this was their final try and should be regarded as their examination score.

### Testing of interest in surgery

During two occasions, when arriving at the test location and when the training session had ended, a questionnaire was completed regarding their interest in surgery in general and minimal-invasive techniques in particular. Subjects rated their interest on a scale 1-4 where 1 corresponded to no interest at all and 4 corresponded to very high interest.

### Testing of motivation

In order to study motivation at the situational level, based on SDT, researchers have developed the Situation Motivation Scale (SIMS).^18^ The scale measures intrinsic motivation with four items (and also identifies regulation, external regulation, and amotivation). Several studies typically have shown that SIMS displays adequate factorial structure and internal consistency.^16^ In response to the item ‘‘Why did you do the simulator training?’’ participants were asked to indicate to what extent each item corresponded to their reason for training, using a 7- point Likert-type scale, ranging from 1 (does not correspond at all) to 7 (corresponds exactly). Sample items for intrinsic motivation include: ‘‘because it is fun’’.

SDT-researchers have used the self-determination index (SDI),^18^ where controlled forms of motivation are subtracted from autonomous, and where regulations are weighted according to their place on a proposed continuum of self-determination. We calculated a SDI for each student. At first scores for each type of motivation were averaged across their respective four items of each sub scale. Each sub scale-score was then weighed according to their position on the continuum and summed by using the following formula: SDI= +2 x (intrinsic motivation) +1 x (identified regulation) -1 x (external regulation) -2 x (amotivation).

Higher scores indicate greater self-determination (autonomy) towards the present simulator training. The SIMS scale was administered after the simulator introduction, after the first simulator and after the last simulator try.

### Statistical analysis

Before designing the study, a power analysis was performed. The primary aim of the study was to evaluate whether intrinsic motivation correlated to performance. It was assumed that the reduction in total score after training would be on average 200 points on the 0-700 point scale, e.g. a reduction from 400 to 200 points. The study was dimensioned in order to detect this difference, with a significance level of 5 % (2-sided), a standard deviation of at most 200 and a power of 80 %. It was also assumed that this reduction in total score would significantly correlate with a raise in motivation measured on a seven-grade scale, 1-7. With 30 subjects these assumptions could be addressed according to the power analysis. 

During the statistical analysis we examined the whole population and also divided subjects into subgroups according to gender and high/low video gaming experience. The reason for doing this was our previous findings in which male subjects generally performed better as well as had a higher video gaming experience.^13^ Statistical comparisons in order to test differences between two independent groups were made by use of the Student’s t-test for uncorrelated means, after validation for normal distribution by use of the Shapiro Wilk´s test. Regression analysis was used in order to evaluate the dependency between variables and the Pearson correlation coefficient was used in order to test independence between variables. In addition to that descriptive statistics was used to characterize the data. All analyses were carried out by use of the SAS system (The SAS system for Windows 9.4, SAS Institute Inc., Cary, NC, USA.) and the 5% levels of significance were considered. In the case of a statistically significant result the probability value (p-value) has been given.

## Results

Regarding the first hypothesis stating that a better performance when completing a surgical training task would correlate with higher intrinsic motivation to train that specific task, we found no significant correlations between the first simulator trial, intrinsic motivation and level of self-determination (derived from the second SIMS test occasion) when analyzing either the total study population, females or groups divided into high/low video gaming experience. There was a significant correlation for male subjects, where a good result in the first simulator trial correlated with a higher SDI (p = 0.05, r = -0.46). Scores for motivation and simulator performance in the groups are shown in Table 1 and 2.

**Table 1:** Scores for background factors and introductory motivation, n=30

Variable	Median	Mean	SD
Age, years	24	25.1	4.6
Computer experience, scale (1-7 low to high)	4	4.1	2.1
Visual-spatial ability, MRT-A test (0-24)	8	7.9	2.4
Introductory interest in surgery (1-4, low to high)	4.3	3.9	0.8
Introductory intrinsic motivation (1-7, low to high)	5.3	5.2	0.7
Introductory self-determination index (-18 - +18, low to high)	10.9	10.8	2.4

Our second hypothesis stated that visual-spatial ability would rank higher in importance to surgical performance than motivation and video gaming experience. There were no significant results in the multivariate analysis with MRT-A score, intrinsic motivation and video gaming experience in relation to simulator results when looking at the total population. The first simulator trial vs. the second SIMS test as well as the best simulator trial and mean simulator performance vs. the last SIMS test occasion were analyzed. Significant results were found in male subjects. Visual-spatial test score was proven to be the most important underlying factor followed by intrinsic motivation score and finally video gaming experience (p = 0.02, p = 0.05, p = 0.11). The same results were true when including SDI instead of intrinsic motivation (p = 0.01, p = 0.01, p = 0.05). Video gaming experience, visual-spatial ability and self-rated interest in surgery are summarized in Table 2.

Our third hypothesis stated that self-determination and intrinsic motivation would correlate with a preference to choose a surgical specialty in the future and simulator training would increase the interest in choosing that same work field. A higher interest in surgery and expressed wish to choose a surgical specialty as a future specialty correlated with a higher level of self-determination, both before (p = 0.03, r = 0.39) and after training (p = 0.03, r = 0.46) when looking at the total population. No similar correlations were detected in the subgroups except among females where a high interest in surgery correlated with a low intrinsic motivation to train before but not after training (p = 0.01, r = -0.74). Interest in surgery increased by simulator training when analyzing all subjects (p = 0.01), male subjects (p = 0.02) as well as subjects with low video gaming experience (p = 0.02).

**Table 2:** MIST-VR simulator performance, total scores*, n=30.

Variable	Median	Mean	SD
First try	396.1	405.6	124.3
Best result entire training period	121.7	136.2	49.5
Average result entire training period	193.1	190.7	62.8

*Range of scores is between 0-700, lower scores indicate better results

The final hypothesis stated that a higher video gaming experience would correlate to a higher intrinsic motivation to train a surgical simulator task. We found a significant correlation between a higher amount of video gaming and a lower amount of intrinsic motivation (p = 0.05, r = -0.48) after the simulator introduction in the subgroup with low video gaming experience when investigating the role of video gaming for motivation to train in the simulator before having tried for the first time. There were no correlations in the other subgroups or the whole population.

To investigate whether there was a correlation between motivation and video gaming in relation to actual training in the simulator; video gaming experience, intrinsic motivation and SDI derived from the second SIMS scale test occasion, i.e. after their first trial, were examined. There was a correlation among females, where a high video gaming experience correlated with a higher intrinsic motivation (p = 0.05, r = 0.6) but a lower SDI (p = 0.01, r = -0.72). The last result proved to be true also among the low video gaming experience subgroup (p = 0.02, r = -0.67).

## Discussion

In this preliminary study we focused on medical students highly motivated for a future career in the field of surgery. We found differences with regards to motivation versus performance as we observed a correlation among male subjects but in no other subgroup, i.e., male students that performed better in the first simulator trial were more motivated to continue training than male students with weak performance in the first simulator trial. Due to the small sample size findings should be interpreted carefully, especially when analyzing subgroups. In order to somewhat compensate for this important limitation, a quite homogenous group of highly motivated medical students were studied. A power analysis was performed when designing the study.  In this study we examined medical students and not residents in surgery, which can be considered a limitation since medical students do not perform image-guided surgery. It would have been more relevant to study surgical residents. Also, since the students were highly motivated, correlations and differences might be harder to detect in such a small sample.

We found that subjects more interested in becoming surgeons experienced a higher level of self-determination during training and that surgical simulator training increased interest in surgery in general. This finding is interesting in relation to Hochberg et. al.´s reference study in which the authors asked 234 residents about their motivation for choosing their field. Fifty-one percent mentioned expected job satisfaction, 44% pointed to intellectual curiosity, and only 3% mentioned prestige, lifestyle or income. Moreover, the majority of surgical residents made their career choices before entering medical school. They became early self-motivated.^19^ Implementing virtual-reality surgical simulator training in the medical students´ basic surgery course may be an effective way of increasing interest in surgery as a future specialty.

There were no positive correlations between simulator training and motivation from a video gaming experience perspective. The results might have been different had we chosen a simulator with anatomical graphics. Considering the high degree of realism and extraordinary graphics of current video games the simulator used in this study (without anatomical graphics) might have been a disappointment in light of the putative expectations. This would be interesting to examine in a future study. The finding gives us additional information about video game training and surgical simulator performance; the positive correlation is unlikely to be explained by higher motivation among video gamers. Instead, video gaming enhances simulator performance due to other properties, possibly such as 3D navigation and eye hand coordination training.

A recent study has shown that distinct learning curves for laparoscopic suturing can be mapped on the basis of visual-spatial ability, psychomotor ability and depth perception. A proportion of subjects with a low degree of the very same abilities were unable to reach proficiency despite repeated attempts.^20^ In the multivariate analysis visual-spatial ability was proven to be more important than motivation for performance, to what extent a high motivation could compensate for lack of visual-spatial ability is left unanswered.

SDT distinguishes between different types of motivation based on the different goals that give rise to a specific action. In order to design training in a motivational way we need to know what aspects of training and background factors alters different types of motivation. High fidelity is not always superior to lower-fidelity because this advantage may be dependent on e.g. the student´s level of competence, the type of task(s), and training curriculum.^21^ Our findings strengthen conclusions from previous research that equipment fidelity is not the only factor that matters; a well-designed curriculum works well with low equipment fidelity and future research may clarify whether high fidelity equipment shortens the learning curve and alters motivation.^22^

In this study we used a single instructor for all subjects. The role and characteristics of the instructor probably plays a great part for skill retention and motivation. Earlier studies have shown for example that video-based coaching enhanced the quality of laparoscopic surgical performance on both virtual-reality and porcine samples, although at the expense of increased time.^23^ Future studies could furthermore investigate the specific role and possible actions of the instructor.

## Conclusions

This preliminary study sought to establish the effect of motivation on surgical simulator performance. Although the investigated sample size is quite small which limits conclusions that can be drawn, we observed individual differences regarding the effect of motivation on simulator performance. This points out the importance of individual customization of surgical training since students are affected differently during their training performance. Whether or not this effect can be altered by instructor feedback could be answered in future studies. However, visual-spatial ability was found to be more important than motivation for performance in the present MIST-VR task, further stressing the importance of innate abilities for simulator training and performance and something to consider in the current debate of how to recruit surgical residents. Furthermore, simulator training increased interest in surgery, which highlights the potential of further utilizing this tool in training of medical students. 

### Acknowledgements

This study was fully supported by unrestricted research grants from the Stockholm County Council and Karolinska Institutet. Drs. Schlickum, Hedman and Felländer-Tsai have no conflicts of interest or financial ties to disclose. Dr. Felländer-Tsai is the director of the Center for advanced medical simulation and training at Karolinska University Hospital. The authors wish to thank Per Näsman for excellent statistical advice.

### Conflict of Interest

The authors declare that they have no conflict of interest.
